# Emerging Role of FAPI PET Imaging for the Assessment of Benign Bone and Joint Diseases

**DOI:** 10.3390/jcm11154514

**Published:** 2022-08-03

**Authors:** Francesco Dondi, Domenico Albano, Giorgio Treglia, Francesco Bertagna

**Affiliations:** 1 Division of Nuclear Medicine, ASST Spedali Civili di Brescia, 25123 Brescia, Italy; f.dondi@outlook.com; 2 Division of Nuclear Medicine, Università degli Studi di Brescia and ASST Spedali Civili di Brescia, 25123 Brescia, Italy; domenico.albano@unibs.it (D.A.); francesco.bertagna@unibs.it (F.B.); 3 Clinic of Nuclear Medicine, Imaging Institute of Southern Switzerland, Ente Ospedaliero Cantonale, 6500 Bellinzona, Switzerland; 4 Department of Nuclear Medicine and Molecular Imaging, Lausanne University Hospital and University of Lausanne, 1015 Lausanne, Switzerland; 5 Faculty of Biomedical Sciences, Università della Svizzera italiana, 6900 Lugano, Switzerland

**Keywords:** PET, FAPI, nuclear medicine, imaging, bone, joint

Positron emission tomography/computed tomography (PET/CT) is a hybrid imaging modality that has recently experienced a wide increase in its use and applications. The most common tracer used to perform such a diagnostic procedure is ^18^F-fluorodeoxyglucose ([^18^F]FDG), which has the ability to reveal hypermetabolic lesions based on their increased glycolytic metabolism. As a consequence, [^18^F]FDG PET/CT has proven its role to assess neoplastic disease both in staging and restaging settings [1,2,3,4,5]. However, its usefulness for the evaluation of inflammatory and infectious diseases has also emerged, in particular in the last decade [6,7,8,9].

In order to evaluate metabolic pathways different from the glucose metabolism, several positron emitters tracers have been proposed as an alternative to [^18^F]FDG. In this setting, fibroblast activation protein (FAP) inhibitors (FAPI), labelled with both ^18^F or ^68^Ga, are emerging as promising radiopharmaceuticals. FAPI are molecules able to bind to FAP, a membrane serine protease that is highly expressed on activated fibroblasts present in a wide range of pathophysiological conditions, such as wound-healing, inflammation and cancer [10,11,12]. In this scenario, the role of FAPI PET/CT for the evaluation of cancer has been evaluated in several studies and the preliminary results are promising, as it also seems to allow the evaluation of tumors with low [^18^F]FDG uptake. Interestingly, when coupled together, [^18^F]FDG and radiolabeled FAPI are able to better define and comprehend tumoral metabolic heterogeneity [10,13,14,15,16].

The role of FAPI imaging is, however, also emerging for the assessment of inflammatory and infectious diseases, even if the evidence in the literature is still in its early stages [10,17,18]. It has been reported that focal radiolabeled FAPI uptake is related to active tissue remodeling in patients with immune-mediated inflammatory diseases that are characterized by the presence of chronic inflammation and tissue response. As a consequence, benign lesions are frequently characterized by the presence of FAPI uptake, and bones or joints are frequently the sites of such findings [18]. Interestingly, inflammatory lesions are not always positive to [^18^F]FDG PET, indicating that inflammation and the related tissue response could be disentangled by the combination of both tracers [19].

The rationale behind the use of FAPI PET/CT for the assessment of inflammatory bone and joint diseases is that FAP is overexpressed in tissue remodeling sites associated with arthritis and fibrosis, such as chondrocytes [10,11]. However, on the other side, some authors hypothesized a different mechanism of uptake, related to the increased vascularity and capillary permeability due to inflammatory response, resulting in high perfusion and blood-pool effects [20]. Nevertheless, one of the advantages of the use of FAPI imaging for the assessment of bone lesions is the fact that bone marrow usually exhibits low physiological FAPI uptake [12,18].

In this scenario, a recent article compared the radiolabeled FAPI uptake in bones and joints between malign and benign lesions (such as periodontitis, osteofibrous dysplasia, degenerative bone disease, arthritis and trauma related lesions) [21]. The authors reported that, even if overlap occurred in some cases, maximum standardized uptake value (SUV_max_) of bone metastases was significantly higher compared to benign lesions, even if no significant differences in terms of uptake between such benign lesions was reported, with the exception of periodontitis. Furthermore, in general, the SUV_max_ of radiolabeled FAPI PET was higher compared to SUV_max_ of [^18^F]FDG PET, with the exception of degenerative disease. The authors also underlined that the features of FAPI-positive benign lesions were the presence of solitary lesions, lower uptake and specific locations, such as the joints.

Several case reports have reported that radiolabeled FAPI uptake is associated with a high number of benign conditions that affect the bones and the joints, such as fibrous dysplasia [22], tuberculosis [23], osteoarthritis, enthesopathies, myositis ossificans [24], Erdheim–Chester disease [25], ankylosing spondylitis [26], fractures [20], synovitis, osteitis and rheumatoid arthritis (RA) [12]. In particular, arthritis is generally underlined as an incidental finding at radiolabeled FAPI PET/CT and the most common sites of uptake are facet, shoulder, sternoclavicular joints and knees [11,20,27,28,29].

Some articles focused particularly on the role of FAPI imaging for the assessment of RA in preclinical setting, with the demonstration of the high specificity of this tracer for arthritic joints, both in vitro and in vivo. In this setting, some pilot clinical evaluations for the use of radiolabeled FAPI PET/CT in subjects with RA have also been proposed, suggesting its potential application to visualize arthritic synovium related to the specific upregulation of the expression of FAP in this site [10,30,31].

The ability of [^18^F]FDG and FAPI imaging to evaluate periprosthetic joint infection (PJI) and aseptic loosening were also compared in preclinical setting, revealing that radiolabeled FAPI PET had higher sensitivity and specificity and had the potential to define more accurately such lesions. Furthermore, FAPI PET had greater potential in the diagnosis of PJI and distinct advantages compared to [^18^F]FDG [32].

To conclude, the role of radiolabeled FAPI PET/CT for the assessment of benign inflammatory and infectious diseases of bone and joints is still in an embryonal phase; however, some insights on its usefulness are emerging. Further data, in particular in clinical settings, are however needed to confirm and better define the advantages of FAPI imaging for the evaluation of such benign lesions, in particular in comparison to [^18^F]FDG.
